# Antibiotic-degrading resistance changes bacterial community structure via species-specific responses

**DOI:** 10.1038/s41396-023-01465-2

**Published:** 2023-06-29

**Authors:** Ayush Pathak, Daniel C. Angst, Ricardo León-Sampedro, Alex R. Hall

**Affiliations:** Institute of Integrative Biology, Department of Environmental Systems Science (D-USYS), ETH Zurich, Zurich, Switzerland

**Keywords:** Microbial ecology, Microbial ecology

## Abstract

Some bacterial resistance mechanisms degrade antibiotics, potentially protecting neighbouring susceptible cells from antibiotic exposure. We do not yet understand how such effects influence bacterial communities of more than two species, which are typical in nature. Here, we used experimental multispecies communities to test the effects of clinically important pOXA-48-plasmid-encoded resistance on community-level responses to antibiotics. We found that resistance in one community member reduced antibiotic inhibition of other species, but some benefitted more than others. Further experiments with supernatants and pure-culture growth assays showed the susceptible species profiting most from detoxification were those that grew best at degraded antibiotic concentrations (greater than zero, but lower than the starting concentration). This pattern was also observed on agar surfaces, and the same species also showed relatively high survival compared to most other species during the initial high-antibiotic phase. By contrast, we found no evidence of a role for higher-order interactions or horizontal plasmid transfer in community-level responses to detoxification in our experimental communities. Our findings suggest carriage of an antibiotic-degrading resistance mechanism by one species can drastically alter community-level responses to antibiotics, and the identities of the species that profit most from antibiotic detoxification are predicted by their intrinsic ability to survive and grow at changing antibiotic concentrations.

## Introduction

Antibiotic resistance is an obstacle to effective treatment of bacterial infections [1, 2]. Strains carrying resistance mechanisms often coexist with other species in multispecies communities. Therefore, to predict responses to antibiotics at the level of individual species or entire communities/microbiomes, we need to understand how resistance mechanisms carried by one species affect neighbouring species in the same community [3, 4]. One common mechanism by which some resistance mechanisms can influence surrounding microbes is by reducing the antibiotic concentration, which can in turn benefit neighbouring susceptible cells [5], sometimes called exposure protection [3, 6]. Important examples of this type of detoxification include carbapenemases and other beta-lactamases [7–11], which enzymatically deactivate beta-lactam antibiotics. Past work showed exposure protection can occur between strains of the same species or pairs of species [3, 6–11]. This suggests detoxification is an important mechanism by which resistance can influence community structure. However, it is not yet known whether or how detoxifying resistance mechanisms influence community structure (relative abundances of different species) in communities of more than two species.

There are multiple ways community structure could change in response to antibiotic degradation. For example, if resident species vary in their ability to grow at degraded antibiotic concentrations, the identities of the species that profit most from detoxification may be predictable from their abilities to grow in pure cultures at relevant concentrations. Depending on the extent of degradation, this may in turn be correlated with interspecific variation of widely measured parameters such as the minimum inhibitory concentration (MIC) or growth rate in the absence of antibiotics [12]. Alternatively, if some of the resident species can become resistant to the extant antibiotic concentration, either via horizontal transfer or other mechanisms, community structure upon detoxification may instead reflect variable rates of resistance evolution. A third possibility is that community-level responses to antibiotic degradation depend on higher-order interactions, such as the growth of some susceptible species in the presence of the resistant species being influenced by interactions with other susceptible species. There is some evidence that higher-order interactions can occur among microbes [13, 14], but no clear consensus about their importance for community structure upon antibiotic exposure [3, 15–17]. Thus, understanding which resident, susceptible species profit most (increase most in relative abundance) when a resistant species detoxifies the local environment would improve our basic understanding of how antibiotic resistance influences microbial diversity, and our ability to predict community-level responses to antibiotics in nature or in treatment contexts.

To test how a potentially detoxifying resistance mechanism affects the relative abundances of different species, we assembled experimental, multispecies communities comprising *Escherichia coli, Staphylococcus aureus, Salmonella enterica* serovar Typhimurium*, Enterococcus faecalis, Pseudomonas aeruginosa* and *Klebsiella pneumoniae*. We chose these species because they are phylogenetically and phenotypically diverse, and all associated with humans, either as commensals [18, 19] or pathogens [20–23]. We tested whether community structure was affected by the carriage of a clinically relevant plasmid encoding a carbapenemase, pOXA-48 [24–26], by one community member (*E. coli*). We monitored plasmid effects on community structure with and without antibiotics, using a clinically relevant combination of piperacillin and tazobactam [27]. Piperacillin is a piperazine derivative of ampicillin [28, 29], generally used in combination with the beta-lactamase inhibitor tazobactam, a penicillanic acid sulfone derivative [30, 31]. We found plasmid carriage by *E. coli* affected community structure via antibiotic degradation, and the species benefitting most from this were identified by their relative abilities to grow at reduced antibiotic concentrations.

## Materials and methods

### Bacteria and growth media

We used seven strains (from six species; Supplementary Table S1) to assemble multispecies communities: *Escherichia coli* K-12 MG1655 (with and without pOXA-48 plasmid)*, Staphylococcus aureus* subsp. *aureus* (Type Strain), *Salmonella enterica* serovar Typhimurium SL1344, *Enterococcus faecalis* JH2-2, *Pseudomonas aerμginosa* PAO1, *Klebsiella pneumoniae* subsp. *pneumoniae* (Schroeter) Trevisan (Type Strain). We cultured bacteria in lysogeny broth (LB) media or on Chromatic MH agar (Liofilchem, Roseto degli Abruzzi, Italy), at 37 °C for 24 h. Each of these species forms colonies with distinct colour/morphology on chromatic agar (Supplementary Fig. S1).

### Plasmid and antibiotics

We used a 63.6-kB pOXA-48-like plasmid from the IncL family carrying a single beta-lactamase gene *bla*_OXA-48,_ conferring resistance to most beta-lactam antibiotics [25]. This plasmid was obtained from a clinical *E. coli* strain isolated in the Division of Clinical Microbiology at University Hospital Basel, Switzerland [32]. This pOXA-48-like plasmid (acc. num. UWXP01000003.1) shares 97% coverage and >99% identity both with the first described pOXA-48 plasmid [33] and with pOXA48_K8, one of the best-studied pOXA-48-like variants [34]. The difference in coverage is primarily due to the lack of a group II intron *ltrA*, suggested to frequently excise/insert [34]. IncL plasmids, and in particular pOXA-48, have been shown to be conjugative and to have a broad host range [34–37]. To test whether the pOXA-48 plasmid used here could be horizontally transferred between bacterial cells, we used a mating assay between the native clinical strain carrying the plasmid and a chloramphenicol-resistant *E. coli* K-12 MG1655 (CmR, Δ*galK::cat*), on agar and in liquid (Supplementary Fig. S2). In our main experiments, we assembled communities with and without the pOXA-48 plasmid by including plasmid-carrying or plasmid-free versions of *E. coli* K-12 MG1655. For communities with antibiotics, we used 7.5 μg/ml piperacillin and 1.5 μg/ml tazobactam, unless stated otherwise. In some experiments, we used selective chromatic agar, including 7.5 or 10 μg/ml piperacillin and 1.5 or 2 μg/ml tazobactam. We used the higher concentrations in some cases to counter the formation of satellite colonies around plasmid-carrying colonies.

### Assembling experimental communities with/without antibiotics and plasmid

To test how the structure of the community was affected by antibiotics and the plasmid, we cultured five replicated communities in four treatments (all combinations of ± antibiotic and ± plasmid). For each replicate microcosm, we first cultivated all constituent species in separate overnight cultures, each inoculated from separate colonies, in 200 μl LB in 96-well microplates. We used independent sets of overnight pure cultures (one per constituent species) for every replicate microcosm. For inoculation of a given microcosm, we diluted the overnight culture of each constituent species 1000-fold into a total volume of 200 μl. Before and after 24 h incubation, we plated dilutions onto chromatic agar to estimate initial and final abundance and community composition. To test for the emergence of antibiotic resistance in susceptible species incubated with the plasmid-carrying strain, we picked all colonies (37 *S*. Typhimurium and 13 *P. aeruginosa*) that survived in any replicate of the antibiotic+plasmid treatment (excluding the plasmid-carrying *E. coli*). We then restreaked these colonies on chromatic agar with piperacillin and tazobactam to check for stable growth at concentrations inhibitory to ancestral strains. As a second test for resistant variants, we plated 20 μl aliquots from all microcosms in all treatments directly onto chromatic agar with piperacillin and tazobactam.

### Liquid chromatography–mass spectrometry (LC-MS)

We tested whether communities incubated with vs without the plasmid (with antibiotics) altered the antibiotic concentration in the growth medium using LC-MS. To prepare samples, we incubated three replicate microcosms as above in the antibiotic+plasmid and antibiotic+no plasmid treatments, and a control treatment (sterile medium with antibiotics). Cultures were prepared as above but with larger volumes (40 ml). We sampled each culture at 0, 6 and 24 h, before filtering (0.2 μm) and freezing (−20 °C). Samples were randomly assigned identifiers before processing to avoid order effects. Gradient separation chromatography was done with an Agilent InfinityLab Poroshell 120 EC-C18 column (150 mm × 2.1 mm, i.d. 1.9 µm; Santa Clara, California, United States) with mobile phase A of 0.1% formic acid in acetonitrile and mobile phase B of 0.1% formic acid in water. The initial mobile phase composition was 1% Phase A up to 1.5 min, followed by a step to 25% Phase A, held for one minute. Within 0.5 min the percentage of phase A increased linearly to 99% and held for 0.5 min. Reconditioning for 2.5 min gave a total run time of 6 min. Column temperature was maintained at 40 °C; injection volume was 10 µl. At these chromatographic conditions, tazobactam and piperacillin-Na have retention times of 2.8 and 3.8 min. Mass spectrometric detection was performed with a Bruker Maxis II ESI-Q-TOF system (Billerica, Massachusetts, United States). Ionisation was achieved using electrospray in the negative ion mode. Spray voltage was set at 3000 V in negative polarity. Nitrogen was used as the nebuliser and dry gas, set at 1.6 bar and 10.0 l/min, respectively. The Vaporiser temperature setting was 250 °C. We expressed measured concentrations as percentages relative to the mean from the control treatment at 0 h (Piperacillin: 5.5 µg/ml, Tazobactam: 2.2 µg/ml; note these values can differ from inoculated concentrations for various reasons, including measurement error and degradation during processing).

### Testing species-specific benefits of antibiotic detoxification on agar

After finding evidence of species-specific effects of detoxification in liquid culture, we made a second test on solid media, where some past work has shown evidence of exposure protection between pairs of strains [8]. We streaked plasmid-carrying *E. coli* across chromatic agar 7.5 µg/ml piperacillin + 1.5 µg/ml tazobactam, then streaked each antibiotic-susceptible strain perpendicularly, with three plates per species. We then checked for evidence growth of the susceptible strain was increased by proximity to the plasmid-carrying strain. To test for such interactions in communities of more than two species, we plated assembled communities of susceptible species onto an antibiotic gradient with a streak of plasmid-carrying *E*. *coli*.

### Antibiotic dose-response curves, survival and growth without antibiotics

To estimate the intrinsic ability of each species to grow at relevant antibiotic concentrations, we tested susceptibility to various concentrations of piperacillin+tazobactam. We prepared each culture as above, inoculating three replicates per strain via 1000-fold dilution, each from an independent overnight culture. We used a two-fold dilution series of piperacillin in LB, ranging from 37.5 to 0.585 μg/ml, plus 0 μg/ml, supplemented with 15 μg/ ml of tazobactam at all concentrations of piperacillin except 0 μg/ml. After 24 h, we measured OD as above. This generated dose-response curves for each species. We also estimated the MIC for each species from these data, taken as the concentration at which there was no detectable growth, using a cut-off for detectable growth of OD ≥0.045. In a separate experiment, we measured the survival of each species, by plating on agar and counting colony-forming units, in pure cultures inoculated as above with 7.5 and 1.5 μg/ml piperacillin and tazobactam (three replicate cultures per species after 0, 2, 4, 6, 24 h).

### Testing for changes in community structure at artificially reduced antibiotic concentrations

We hypothesised artificially reducing the antibiotic concentration would result in similar community composition in microcosms without plasmid-carrying *E. coli* compared to communities from the antibiotic+plasmid treatment of the main experiment. That is, we aimed to mimic the detoxifying effect of the plasmid by extraneously reducing the antibiotic concentration. We inoculated four replicate communities as above, without the plasmid-carrying strain, at various antibiotic concentrations (7.5 + 1.5, 0.9 + 0.18, 0.46 + 0.09, 0.23 + 0.04, and 0 + 0 μg/ml of piperacillin+tazobactam, equivalent to 100, 12.5, 6.25, 3.125 and 0% of the concentrations in the main experiment). After incubation, we inferred community structure and abundance by plating as above.

### Pairwise interactions between plasmid-carrying *E. coli* and other species

We tested whether species benefiting most from detoxification in the main experiment above also benefited most from detoxification in two-species cultures with only the plasmid-carrying *E. coli*. In other words, we asked whether the benefits of detoxification were specific to six-species communities (e.g., because they rely on higher-order interactions), or if they could be explained by pairwise interactions between individual susceptible species and the plasmid-carrying *E. coli* strain. We cultivated three replicates of each species in pure cultures (without antibiotics), and in co-cultures with plasmid-carrying *E. coli* (with and without antibiotics). We prepared cultures as above, inoculating by 1000-fold dilution from overnight cultures of each species (that is, the total number of cells was higher in the two-species cultures than in the pure cultures). This set-up tests whether the growth of each species is altered by the addition of plasmid-carrying *E. coli* relative to how it grows on its own when inoculated at the same density, following past work [38, 39]. We then incubated and plated as above, including a separate plating on piperacillin+tazobactam to test for resistance evolution.

### Statistical analysis

**Fig. 1:**
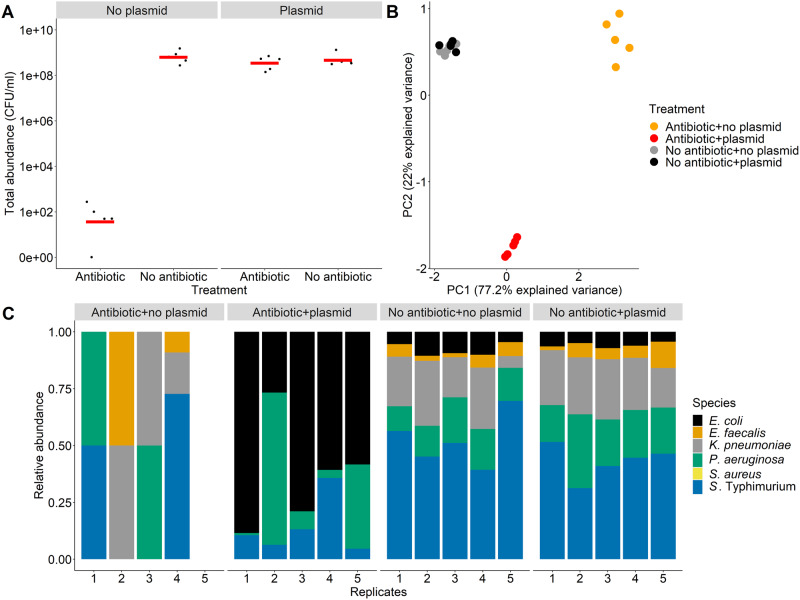
Community abundance and structure with/without antibiotics and a resistance plasmid. **A** Estimated total abundance (CFU/ml) in five replicated microcosms in each treatment (with/without a resistance plasmid carried by *E. coli*, and with/without antibiotic treatment with piperacillin+tazobactam). **B** Principal component analysis of community structure in the different treatments; input variables are absolute abundances of the six species in each microcosm. **C** Community structure in different treatments; estimated relative abundances of each species in five replicate microcosms in each treatment. In treatments with plasmids, the plasmid was carried by *E. coli* and each microcosm contained only one *E. coli* strain (either with plasmid or without depending on the treatment).

**Fig. 2:**
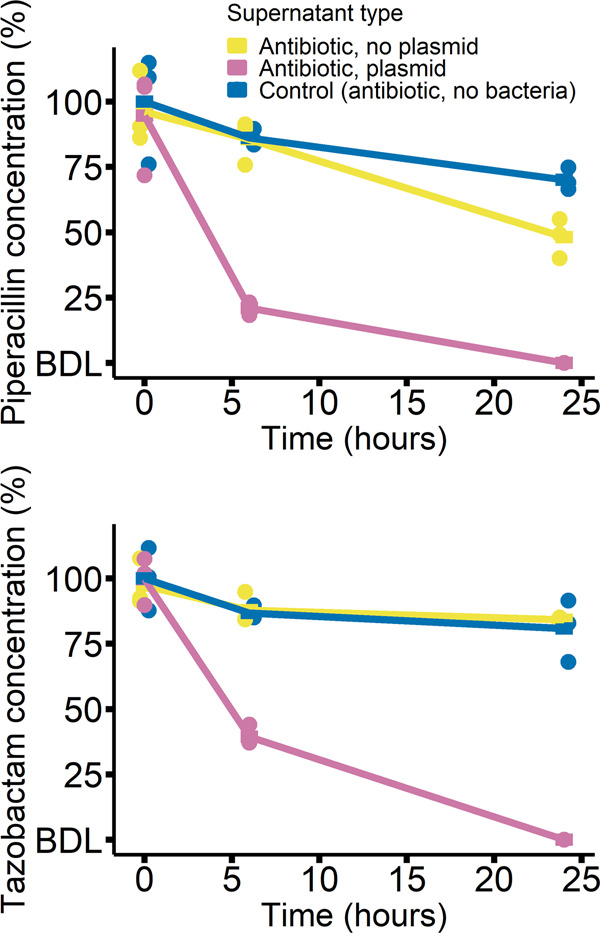
Piperacillin and tazobactam concentrations measured by LC-MS in supernatants from community cultures with and without plasmid, or sterile medium, after 0, 6 and 24 h of incubation. Concentrations are shown relative to the mean 0 h concentration in the control treatment (see Methods). Bars represent means (*n* = 3) and “BDL” signifies below detection limit for the compounds.

Experiments associated with Figs. 1, 2, 5, and 6 were randomised and analyses were conducted in R 4.1.0. To analyse community structure, we used principal component analysis of absolute abundances (CFU/ml) of each species in the main experiment. Variables were shifted to be zero centred and scaled to have unit variance. We also used permutational multivariate analysis of variance (permANOVA) based on Bray–Curtis dissimilarity matrix, using the “vegan” R package [40, 41]. To estimate pure-culture growth rates without antibiotics, we used the “fitR” package [42]. This uses a sliding-window approach to estimate the maximum slope of OD over time for a defined number of consecutive points (here, five consecutive points with intervals of 15 min).

## Results

### Resistant *E. coli* rescues *P. aeruginosa* and *S*. Typhimurium from antibiotic inhibition

When we cultivated experimental multispecies communities with antibiotics, inclusion of a resistance plasmid in *E. coli* reduced the effect of antibiotics on total bacterial abundance (Fig. 1A, two-way ANOVA, plasmid × antibiotic interaction: *F*_1,16_ = 229.5, *p* < 0.001). Community structure, in terms of relative abundances of different species (Fig. 1B, C), was also significantly affected by both antibiotics and by the plasmid (permANOVA, effect of plasmid: pseudo-*F*_1,16_ = 14, *p* = 0.001; effect of antibiotic: pseudo-*F*_1,16_ = 16.3, *p* = 0.001). In the absence of the plasmid, antibiotic treatment resulted in high variability among replicate microcosms, and fewer species being detected at the end of the experiment (Fig. 1C). Total bacterial abundance in these microcosms (with the antibiotic, without the plasmid) was lower after 24 h than at 0 h, indicating community structure here reflected a small fraction of surviving cells from some species, rather than variable population growth across the plasmid-free species (mean total abundance after 24 h = 95 CFU/ml, SD = 106.65, mean at 0 h = 2.2 × 10^6^ CFU/ml, SD = 1.3 × 10^6^). The plasmid had little effect on community composition in the absence of antibiotics, but upon antibiotic treatment it enabled *P. aeruginosa*, *S*. Typhimurium and *E. coli* to grow (antibiotic × plasmid interaction in permANOVA: pseudo-*F*_1,16_ = 14.2, *p* = 0.001; Fig. 1C). Thus, as well as increasing growth of the strain carrying the plasmid (*E. coli*) in the presence of antibiotics, the plasmid rescued some otherwise susceptible community members (*P. aeruginosa* and *S*. Typhimurium) from antibiotic inhibition.

### No evidence of resistance evolution in antibiotic-susceptible species

Variation among the susceptible species in terms of their abilities to acquire antibiotic resistance during the experiment is a potential explanation for why some species performed better than others in the presence of plasmid-carrying *E. coli* plus antibiotics. We tested this by restreaking colonies from each of the initially susceptible species from the end of the main experiment onto antibiotic agar. We picked all *S*. Typhimurium and *P. aeruginosa* colonies (37 *S*. Typhimurium colonies and 13 *P. aeruginosa* colonies) from five chromatic agar plates from the antibiotic+plasmid treatment, and restreaked them on chromatic agar supplemented with antibiotics. None of the restreaked colonies formed new colonies on antibiotic agar. As a second test, we plated 20 μl aliquots from all microcosms of the four treatments (Fig. 1) on chromatic agar plates with 10 μg/ml piperacillin and 2 μg/ml tazobactam. On these plates, the only colonies we observed were of *E. coli* with the pOXA-48 plasmid. Thus, we found no evidence that the rescue of susceptible species caused by the addition of the plasmid in the presence of antibiotics (observed above) was linked to the acquisition of resistance during the experiment. Despite this, a separate mating experiment between the native clinical donor strain and *E. coli* MG1655_CmR showed the pOXA-48 plasmid was conjugative at high rates in other experimental conditions (Supplementary Fig. S2), as previously reported for other variants [34].

### Plasmid carriage by *E. coli* detoxifies the environment for other species

Measuring antibiotic concentrations in growth medium extracted from community treatments over time showed plasmid carriage by *E. coli* greatly accelerated the decline in piperacillin and tazobactam concentrations (Fig. 2). We investigated the downstream effects of such changes for the growth of susceptible species, by culturing each species individually in supernatant from communities incubated as in the main experiment in the antibiotic + plasmid treatment, the antibiotic + no plasmid treatment, and sterile medium with antibiotics (Supplementary Fig. S3). For all susceptible species (species without the plasmid), population growth was higher in supernatants from communities with the plasmid than without (Supplementary Fig. S3). The magnitude of this effect varied among the susceptible species (two-way ANOVA with growth in undiluted supernatant as a response variable, excluding control supernatant, species × supernatant type interaction: *F*_5,24_ = 26.4, *p* < 0.01). *P. aeruginosa*, *K. pneumoniae*, *S. aureus* and *S*. Typhimurium all showed relatively strong responses to detoxification (difference between plasmid+ and plasmid-supernatant types) compared to *E. faecalis* and plasmid-free *E. coli*. Note other changes to the growth medium besides the antibiotic concentration, such as altered nutrient status or other effects of bacterial growth [43], could potentially also have species-specific effects in subsequent cultures. Both types of community-derived supernatant (with the plasmid and without) supported more growth than the control supernatant without bacteria, indicating there was some antibiotic detoxification even without the plasmid (Supplementary Fig. S3), for example, due to interactions with target cells or the inherent instability of piperacillin [44, 45]. In summary, communities with the plasmid detoxified the abiotic environment via degrading the antibiotics, but some species were better than others at exploiting this.

### Species-specific benefits of antibiotic degradation on agar

**Fig. 3:**
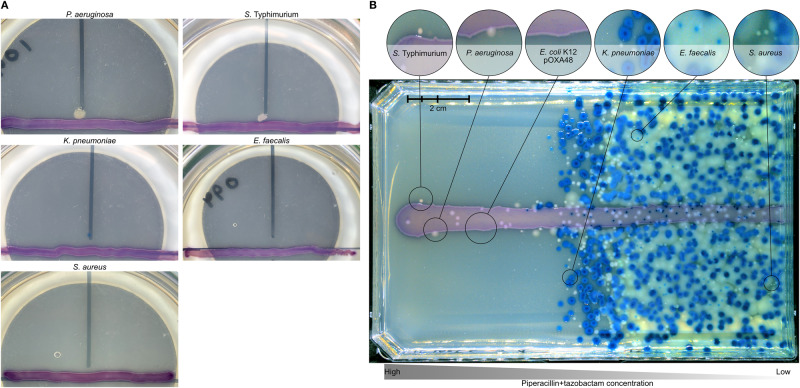
Species-specific benefits of detoxification on agar. **A** Two out of five antibiotic-susceptible species (*P. aeruginosa* and *S*. Typhimurium) show visible growth on antibiotic agar in proximity to a resistant, plasmid-carrying *E. coli* strain. Each susceptible species was streaked on chromatic agar with antibiotics (vertical black line) perpendicular to the pOXA-48 carrying *E. coli* (purple horizontal line) and incubated for 24 h. Each combination was replicated three times and one replicate is shown; other replicates were similar in all combinations (three out of three for *P. aeruginosa* and two out of three for *S*. Typhimurium were qualitatively identical; see Supplementary Fig. S4. **B** Multispecies communities plated on a gradient of piperacillin+tazobactam (left-to-right; maximum concentration in overlay agar = 5 µg/ml piperacillin and 1 µg/ml tazobactam) with the plasmid-carrying *E. coli* strain streaked horizontally (Supplementary Fig. S5).

As a second test for species-specific benefits of detoxification by the plasmid-carrying strain, we streaked all antibiotic-susceptible species from the antibiotic+plasmid treatment of the main experiment perpendicularly to the plasmid-carrying *E. coli* on chromatic agar with antibiotics (Fig. 3A and Supplementary Fig. S4). After incubation, only *P. aeruginosa* and *S*. Typhimurium showed visible growth, and only in proximity to the plasmid-carrying strain. In a further test for interactions on agar, but at the community level, we plated entire multispecies communities on agar with a gradient of piperacillin+tazobactam, with the plasmid-carrying *E*. coli streaked across the middle (Fig. 3B). This showed again that *P. aeruginosa* and *S*. Typhimurium were among the species best able to exploit detoxification (colonies on the high-antibiotic side of the plate in proximity to the plasmid-carrying strain; Fig. 3B and Supplementary Fig. S5). This supports the species-specific benefits of detoxification we observed above in liquid culture, and shows this can also apply in a spatially structured environment.

### Species that profit most from detoxification also grow relatively well at reduced antibiotic concentrations in pure culture

**Fig. 4:**
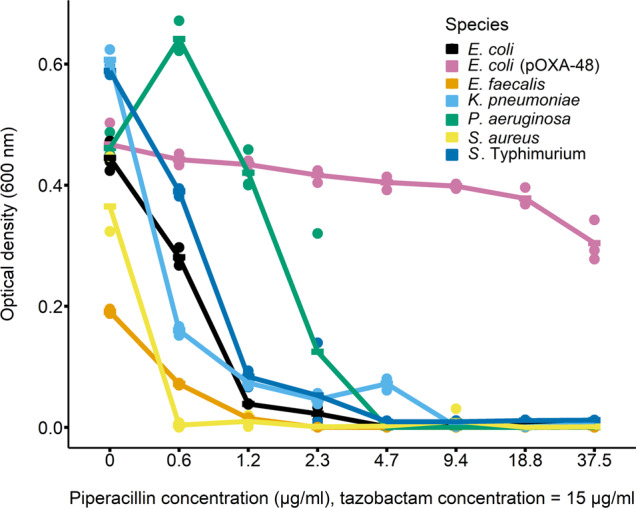
Dose-response curves for all species with varying concentrations of piperacillin and a constant tazobactam concentration. Bars represent means (*n* = 3).

One possible explanation for species-specific benefits of detoxification is that some species grow better than others at reduced antibiotic concentrations. The relevant range of concentrations in our experiments includes 7.5 μg/ml (the starting concentration used in the main experiment) and non-zero concentrations lower than 7.5 μg/ml. In pure culture, no species except the plasmid-carrying *E. coli* showed significant growth at 7.5 μg/ml piperacillin or higher (Fig. 4). At non-zero concentrations lower than 7.5 μg/ml, *P. aeruginosa* and *S*. Typhimurium were the best-performing susceptible species at all but one of the tested concentrations (Fig. 4). Thus, the species that profited most from antibiotic detoxification by plasmid-carrying *E. coli* in our main experiment (Fig. 1), in supernatant assays (Supplementary Fig. S3), and on agar-plate assays (Fig. 3), were those that grew best in pure cultures at reduced antibiotic concentrations. Variation among susceptible species in terms of growth at reduced concentrations was not simply predicted by their relative MICs (inferred from the dose-response curves shown in Fig. 4): *Klebsiella pneumoniae* (MIC: 9.375 μg/ml) had a higher MIC than *P. aeruginosa* (MIC: 4.65 μg/ml) and *S*. Typhimurium (MIC: 4.65 μg/ml) but performed worse at most non-zero concentrations lower than 7.5 μg/ml. The relatively high MIC of *K. pneumoniae* also agrees with its dense growth at higher antibiotic concentrations compared to other species on agar (Fig. 3B).

Unlike growth at reduced antibiotic concentrations, the relative abilities of different species to grow in the absence of antibiotics did not explain the success of *P. aeruginosa* and *S*. Typhimurium in the antibiotic+plasmid treatment of the main experiment. This is shown firstly by another species (*K. pneumoniae*) reaching abundances similar to or greater than *P. aeruginosa* in plasmid-free, antibiotic-free microcosms of the main experiment, but being excluded in the antibiotic+plasmid treatment (Fig. 1). It is also possible that relative final abundances in community microcosms with antibiotic and the plasmid depend more on the intrinsic growth rates of individual species during exponential phase without antibiotics, rather than on their relative final abundances without antibiotics as measured above (Fig. 1). To investigate this, we estimated the maximum growth rate in the absence of antibiotics for each species (Supplementary Fig. S6). This showed, for both *P. aeruginosa* and *S*. Typhimurium, there was at least one other antibiotic-susceptible species with a similar or higher maximum growth rate in the absence of antibiotics (Supplementary Fig. S6; effect of species in one-way ANOVA: *F*_6,14_ = 12.4, *p* < 0.05, with Tukey’s HSD comparisons). Final abundances in the same pure-culture assays supported a similar conclusion (Supplementary Fig. S6).

An additional possible contributor to the success of *P. aeruginosa* and *S*. Typhimurium in multispecies microcosms is variable survival, rather than growth, upon exposure to antibiotics during the initial phase before degradation. In a time-resolved killing assay over 24 h in pure cultures (Supplementary Fig. S7), we found *P. aeruginosa* and *S*. Typhimurium showed relatively high survival compared to *E. coli*, *E. faecalis* and *K. pneumoniae* at some time points, particularly over the first few hours. *S. aureus* also showed relatively high survival. Thus, relatively high survival (a lower proportional reduction in CFU/ml over time) may also have contributed to the success of *P. aeruginosa* and *S*. Typhimurium in our main experiment.

### Artificially reducing the antibiotic concentration reproduces some of the same dynamics as observed upon plasmid introduction

**Fig. 5:**
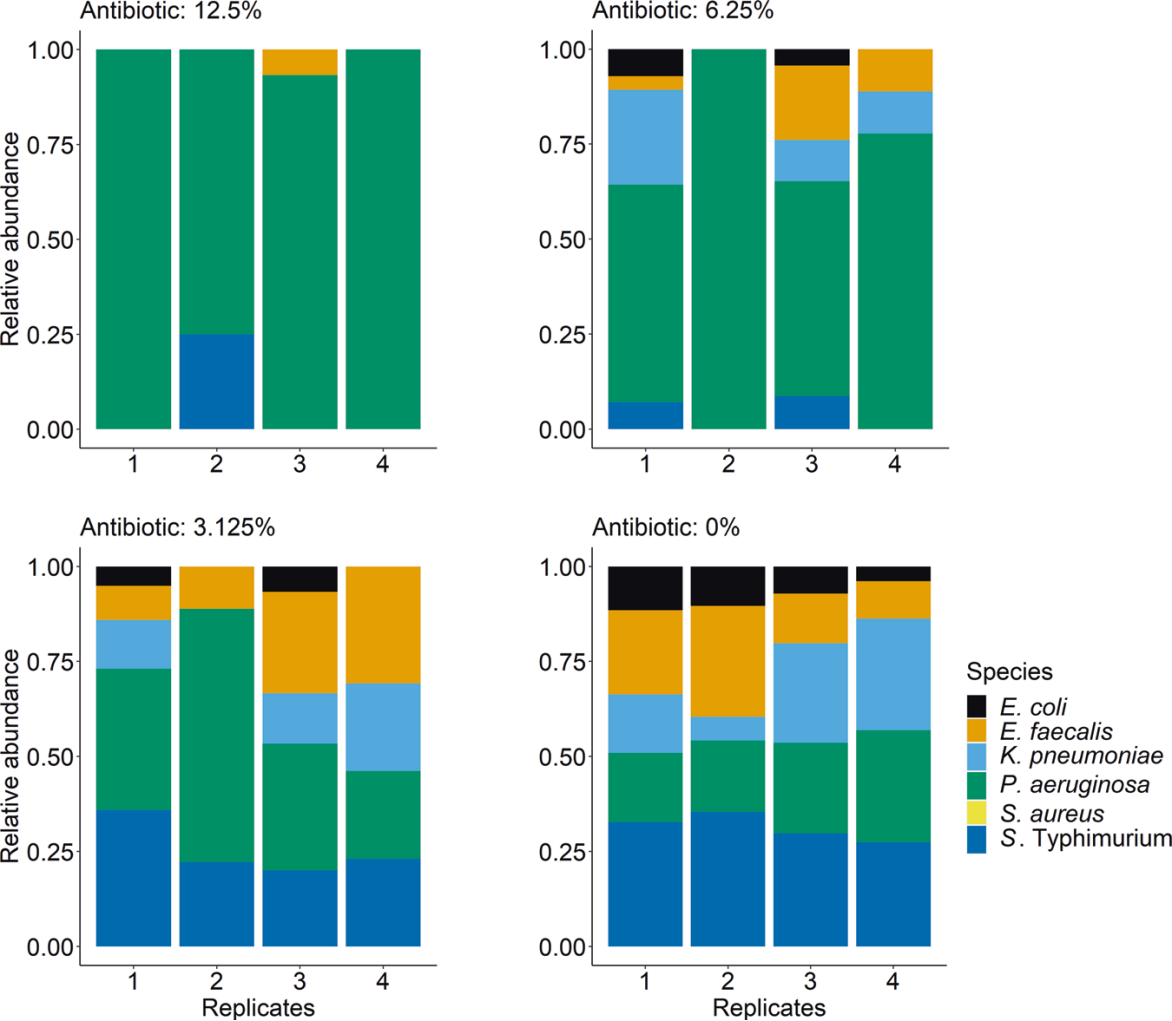
Community structure in microcosms without plasmid-carrying *E. coli* at various starting concentrations of antibiotics. Each panel shows a different antibiotic concentration given as a percentage of the starting concentration in the main experiment (Fig. 1): 12.5% = 0.9 μg/ml piperacillin and 0.18 μg/ml tazobactam, 6.25% = 0.46 μg/ml piperacillin and 0.09 μg/ml tazobactam, 3.125% = 0.23 μg/ml piperacillin and 0.04 μg/ml tazobactam, 0% = No antibiotic. The 100% treatment (7.5 μg/ml piperacillin, 1.5 μg/ml tazobactam) is not shown; we detected negligible growth in this treatment, with 3 out of 4 replicates having no colonies at all.

We hypothesised our results were driven by plasmid-encoded resistance detoxifying the local abiotic environment. If this is true, artificially reducing the concentration of antibiotic, even in the absence of plasmid-encoded resistance, should result in some of the same changes in community structure as observed in the antibiotic + plasmid treatment of the main experiment. Without antibiotics, as expected, community structure resembled that of the antibiotic-free communities from our main experiment (Fig. 5, 0%). At intermediate concentrations (greater than zero but lower than that used in the main experiment), *P. aeruginosa* was the most abundant susceptible species. *S*. Typhimurium also performed well in some microcosms at all intermediate concentrations tested. This supports our other results indicating these two species profit most from plasmid-mediated detoxification. We nevertheless interpret this experiment with caution, because changing the starting concentration does not recapitulate all possible effects in our main experiment, such as the temporal dynamics of antibiotic degradation or plasmid effects on the growth of its *E. coli* host.

### Higher-order interactions are not required to explain species-specific benefits of detoxification

**Fig. 6:**
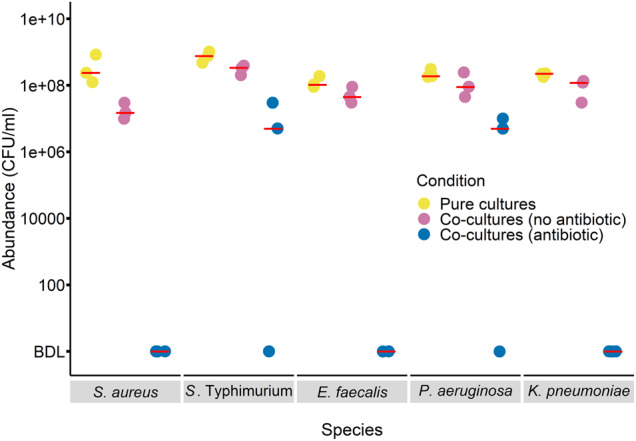
Only *P. aeruginosa* and *S*. Typhimurium show viable population growth in co-cultures with plasmid-carrying *E. coli* plus antibiotics. Abundance (CFU/ml) of each antibiotic-susceptible species excluding *E. coli* after 24 h incubation is shown for three replicates in pure culture without antibiotics, in pairwise co-culture with the plasmid-carrying *E. coli* without antibiotics, or in pairwise co-culture with the plasmid-carrying *E. coli* with antibiotics. The red bars represent the median values for each condition. Note the detection limit in the antibiotic treatment here is quite high (5 × 10^6^ CFU/ml; below detection limit is signified as “BDL” on *y*-axis), due to the density of plasmid-carrying *E. coli* in those cultures.

The above experiments suggest *P. aeruginosa* and *S*. Typhimurium profit most from detoxification because they grow better than other species at degraded antibiotic concentrations, and therefore higher-order interactions are not required to explain changes in community structure upon detoxification. In this case, we would expect to see similar patterns as in the main experiment when each susceptible species is incubated only with the plasmid-carrying *E. coli* strain (without other susceptible species). We found this to be the case: in co-cultures with the plasmid-carrying strain plus antibiotics, only *S*. Typhimurium and *P. aeruginosa* reached detectable population densities (Fig. 6). As in our main experiment, plating on antibiotic agar revealed no evidence of resistant variants of susceptible species. In the same experiment we included treatments without antibiotics, both in pure and co-cultures. In both these treatments, some of the other species performed similarly well compared to *P. aeruginosa* and *S*. Typhimurium (Fig. 6; effect of species in one-way ANOVA: *F*_4,10_ = 4.8, *p* < 0.05 for pure cultures and *F*_4,10_ = 7.7, *p* < 0.05 for co-cultures without antibiotics; Tukey’s HSD: *p* > 0.05 for multiple pairwise comparisons between both *P. aeruginosa* and *S*. Typhimurium and the other species in both treatments). This experiment therefore indicates, as above, *P. aeruginosa* and *S*. Typhimurium profited most from detoxification, even when the other susceptible species were absent, and this was not associated with superior antibiotic-free growth of these two species.

## Discussion

In six-species communities, *E. coli* carrying a resistance plasmid detoxified the environment for other species. This had large effects on community structure, because some species profited more than others from detoxification. The identities of the species profiting most were explained by interspecies variation of their intrinsic population growth (measured in pure-culture) at reduced antibiotic concentrations (lower than the starting concentration). By contrast, two widely measured parameters in resistance evolution (MIC and pure-culture growth rate without antibiotics) only partially explained the success of *P. aeruginosa* and *S*. Typhimurium in detoxified community microcosms. Further experiments all supported a similar pattern in terms of how detoxification changed community structure. The two species profiting most also had relatively high survival compared to most other species during the initial high-antibiotic phase, suggesting the variable capacity for survival, as well as population growth, influences community-level responses. We found no evidence that direct or higher-order interactions among susceptible species influenced community-level responses to detoxification, despite evidence of these in some other scenarios [13, 14, 46]. Thus, our results reveal community-level effects of detoxifying resistance mechanisms, including which species are most likely to increase in relative abundance upon antibiotic degradation.

The first important implication of our results is that the shift in community composition we observed upon plasmid-mediated detoxification was better explained by interspecies variation of the capacity to grow at degraded antibiotic concentrations than by classically measured parameters, including MIC and antibiotic-free growth rate. *P. aeruginosa* and *S*. Typhimurium grew better than other species at non-zero concentrations below the starting concentration, and were the only two species successfully exploiting detoxification in community treatments in our main experiment. These two species also had relatively high MICs, but so did *K. pneumoniae* (both in liquid and on agar), despite *K. pneumoniae* being unsuccessful in detoxified community microcosms. Thus, we do not argue that the MIC is uninformative, but it did not fully explain which species profited most in our experiments. Similarly, other species had similar growth rates in the absence of antibiotics compared with *P. aeruginosa* and *S*. Typhimurium, but did not thrive in detoxified community microcosms. This suggests, when there are detoxifying resistance mechanisms present, responses of individual species depend critically on how they respond to degrading concentrations, not only their antibiotic-free growth rates and MICs [3, 47]. In future work with greater numbers of species, interpretation of this type of interspecific variation could be facilitated by pharmacodynamic modelling [48]. Our data also suggest interspecies variation of survival during the initial phase of exposure to high-antibiotic concentrations contributed to the success of *P. aeruginosa* and *S*. Typhimurium. Survival of biocidal concentrations has been reported previously as a determinant of exposure protection [8]. Overall, this supports initial survival then growth at degrading concentrations as drivers of individual species’ responses.

Our findings are likely to apply in other scenarios. First, the pOXA-48 plasmid is widespread and highly clinically relevant [49], so understanding this particular plasmid is important in itself. Second, the mechanism by which detoxification is encoded on this plasmid, via an antibiotic-degrading beta-lactamase enzyme, is common among other plasmids, bacteria and resistance genes [3, 8, 10]. Third, the species in our communities are common in nature and associated with humans as commensals and/or pathogens. Moreover, the principle mechanism by which community composition changed in our experiment relies on interspecific variation of responses to antibiotic degradation, which we expect to be pervasive also in natural communities. Fourth, we found similar patterns in terms of species-specific responses to detoxification in liquid and on agar, indicating this also applies in spatially structured conditions. An interesting avenue for future work would be to investigate spatio-temporal dynamics of community growth relative to antibiotic exposure in more detail, such as whether biofilm formation prior to exposure permits more species to profit from detoxification.

We found no evidence of a role for horizontal plasmid transfer in community-level responses to detoxification, despite the pOXA-48 plasmid’s capacity for transfer into at least one of the susceptible strains [34, 50]. We do not rule out the possibility of transfer in our experiments, but even if this occurred, it did not explain which species profited from detoxification (neither *P. aeruginosa* nor *S*. Typhimurium detectably acquired resistance). Our results are therefore also relevant for scenarios where detoxifying resistance is encoded on non-mobile elements. Nevertheless, future work testing the transfer dynamics to the other susceptible species in other conditions would be informative. In addition, we speculate detoxification itself may contribute to a reduced role for acquisition of resistance by susceptible species (via horizontal transfer or by chromosomal mutation) compared to scenarios where resistant species do not degrade the antibiotic. This is because detoxification should reduce the selective advantage of any newly arising resistant variants relative to their susceptible counterparts (we expect this advantage to be greatest above the MICs of susceptible strains but below the MIC of the resistant strain), resulting in a slower increase in transconjugant frequency. Nevertheless, we do not exclude the possibility of surviving susceptible species eventually evolving resistance in communities including a detoxifying strain, and this could be tested by experiments over longer timescales. Such longer-term, evolutionary responses to antibiotics may also alter interactions among the susceptible species, as observed recently between *P. aeruginosa* and *Stenotrophomonas maltophilia* [46].

In conclusion, carriage of a representative antibiotic-degrading resistance mechanism by one species changed the responses of neighbouring species to antibiotic exposure in multispecies communities. This caused a shift in community composition and total abundance relative to equivalent communities without resistance. The extent to which susceptible species benefited from antibiotic degradation depended on their intrinsic ability to survive and then grow at changing antibiotic concentrations. This shows responses of individual species to antibiotics can depend on resistance mechanisms circulating in their neighbours. As clinical metagenomic data becomes more widely available and applicable in treatment contexts and diagnostics [51], annotation of potentially degrading resistance mechanisms such as beta-lactamases in microbiome samples, even if they are not detected in targeted pathogens, may prove informative. The relevance of this is further supported by evidence antibiotic-degrading mechanisms, like in our experiments, are common in human-associated communities [52].

## Supplementary information

Antibiotic-degrading resistance changes bacterial community structure via species-specific responses

## Data Availability

The datasets generated during and/or analysed during the current study are available in the dryad repository, digital object identifier (DOI): 10.5061/dryad.63xsj3v7k.
