# Supplementary material for: Antibiotic-degrading resistance changes bacterial community structure via species-specific responses
Source: ISME J. 2023 Jun 29;17(9):1495–503. doi: 10.1038/s41396-023-01465-2 (PMC10432403; doi:10.1038/s41396-023-01465-2)
Supplement: Supplementary file 1 — Antibiotic-degrading resistance changes bacterial community structure via species-specific responses [file 41396_2023_1465_MOESM1_ESM.docx]

**Supplementary Material**

Antibiotic-degrading resistance changes bacterial community structure via species-specific responses

Ayush Pathak^1^, Daniel C. Angst^1^, Ricardo León-Sampedro^1^, Alex R. Hall^1^

^1^ Institute of Integrative Biology, Department of Environmental Systems Science (D-USYS), ETH Zurich, Zurich, Switzerland

# Contents

Table S1: Description of bacterial strains

Figure S1: Pure culture colonies of all species used in this study on chromatic agar.

Figure S2: Conjugative transfer of pOXA-48 plasmid from native clinical isolate to K-12 MG1655

Figure S3: Supernatant growth assays of bacterial strains

Figure S4: Growth dynamics of bacterial strains

Figure S5: Species-specific benefits of detoxification on agar. Images of antibiotic exposure protection of susceptible strains on agar.

Figure S6: Images of antibiotic exposure protection of assembled communities on agar.

Figure S7: Decline in species-specific pure culture abundances due to antibiotic exposure over time.

**Table S1.** Description of all the strains used in this study. ATCC refers to American Type Culture Collection.

| Species | Strain | Citation or ATCC reference |
| --- | --- | --- |
| *Escherichia coli* | K-12 MG1655 Δ*galK::ca*t (CmR) | (1) |
| *Escherichia coli* | K-12 MG1655 Δ*galK::cat* (CmR, pOXA-48) |  |
| *Staphylococcus aureus* | Type Strain | ATCC 12600 |
| *Salmonella enterica* serovar Typhimurium | SL1344 | ATCC SL1344 |
| *Enterococcus faecalis* | JH2-2 | (2) |
| *Pseudomonas aeruginosa* | PAO1 | (3,4) |
| *Klebsiella pneumoniae* subsp. *pneumoniae* | Type Strain | ATCC 13883 |

**
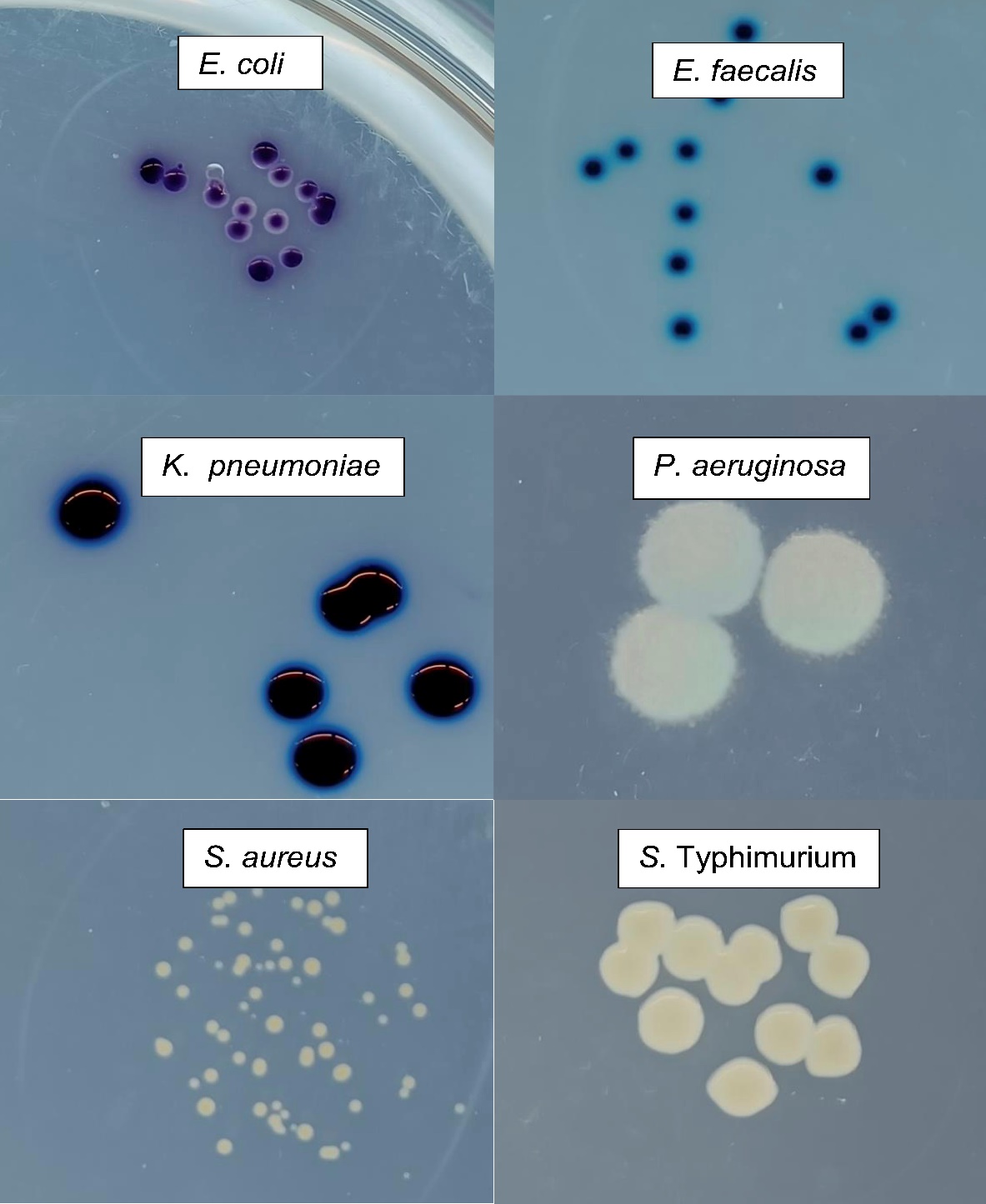
**

**Figure S1: Colonies from pure cultures of all species on chromatic agar.** Colonies are shown after 24 h of incubation. Pictures are not to scale. In practice, *S.* Typhimurium and *S. aureus* are also differentiated on the basis of opacity (*S. aureus* colonies are more opaque than *S.* Typhimurium colonies), and *K. pneumoniae* and *E. faecalis* colonies are differentiated on the basis of both color and shape (*K. pneumoniae* colonies are more convex and darker compared with *E. faecalis* colonies).

**
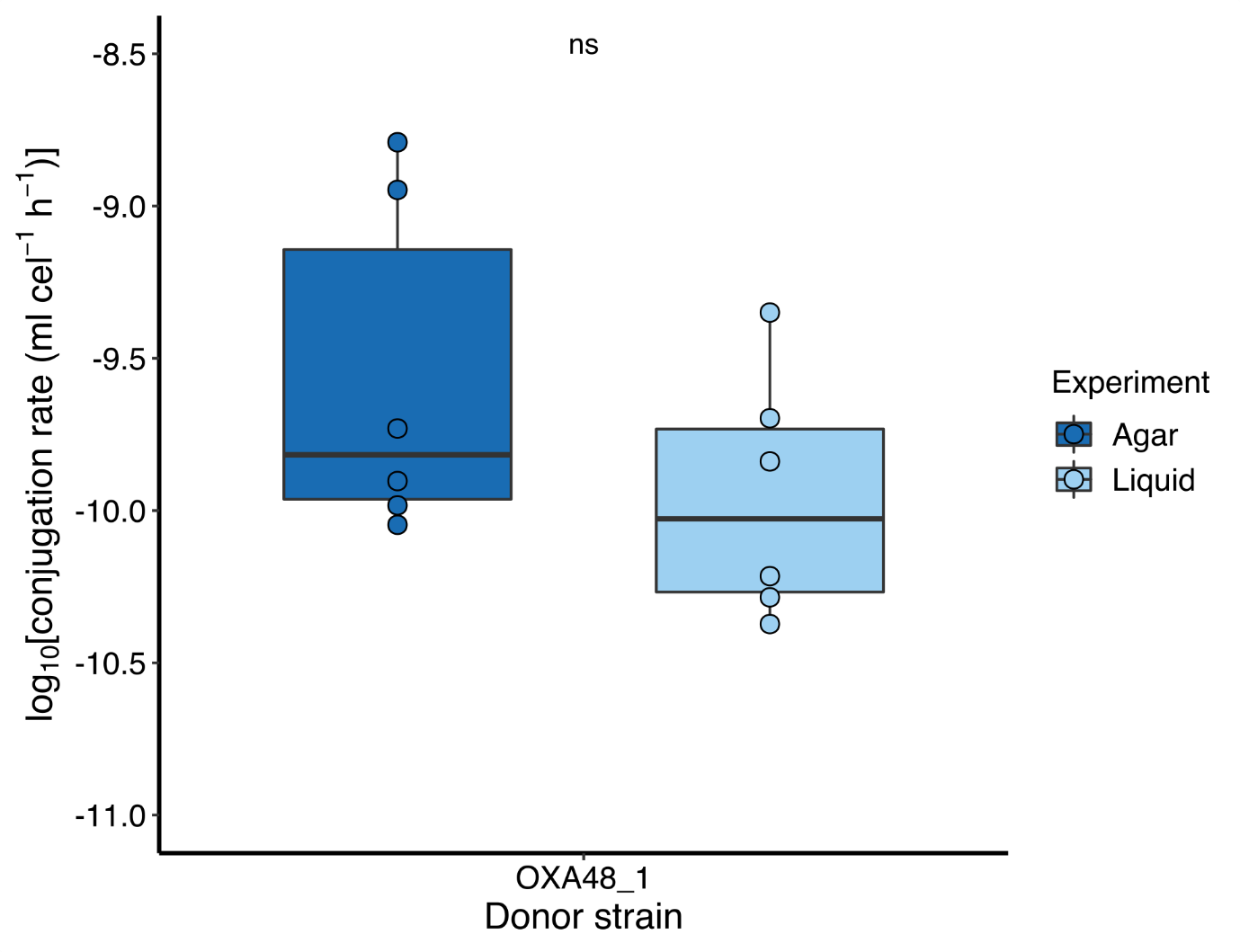
Figure S2: Conjugation rate of** **pOXA48 into a chloramphenicol resistant *E. coli* K-12 MG1655 Δ*galK::cat* on agar and in liquid** (5,6). For the agar assay, we first grew six colonies each of the *E. coli* clinical strain (native plasmid host) and the recipient (MG1655_CmR) in 2 ml of LB. After 3.5 h at 37 °C with 180 r.p.m., we pelleted each culture (1 ml centrifuged 5 min at 1500G), resuspended in 100 µl LB, then mixed donor:recipient cultures 1:1 (*v*:*v*) and spotted onto LB agar plates. After 1 h at 37°C, spots were resuspended in sterile NaCl 0.9% and appropriate dilutions plated on selective plates (ampicillin 100 mg l^−1^, chloramphenicol 50 mg l^−1^ and a combination of both). For the liquid assay, after mixing the independent overnight cultures of the donor and recipient in a 1:1 ratio, we incubated a 1000-fold dilution for 24 h at 37°C. After 24h, appropriate dilutions were plated on selective plates. Plasmid presence was verified by *bla*_OXA-48_ gene amplification by PCR (Fw: GGCGTAGTTGTGCTCTGGAA; Rv: CCAACCGACCCACCAGCCAA). Conjugation rates were determined using the end-point method (7).

**
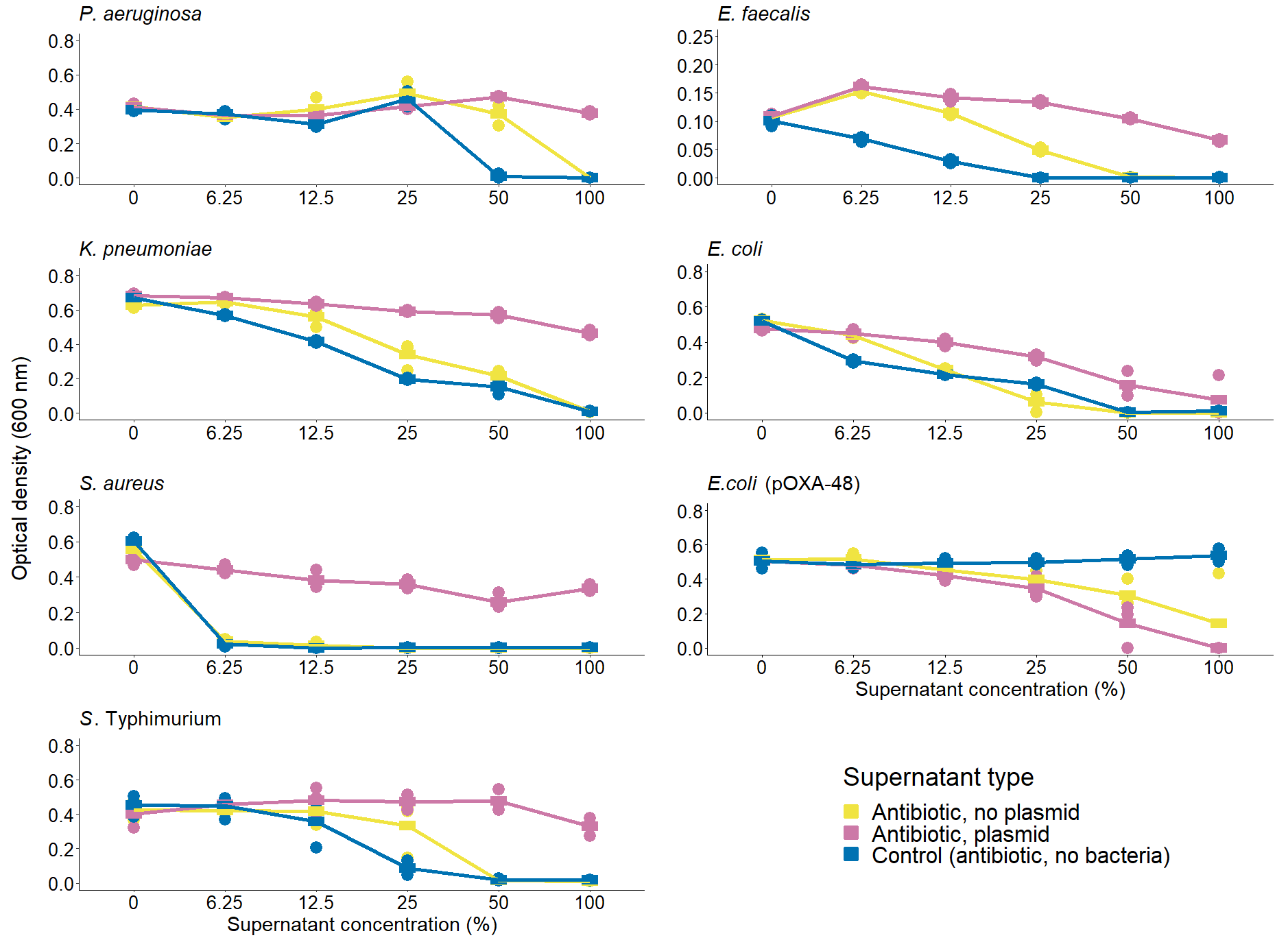
**

**Figure S3.** Abundance (optical density at 600 nm) of each species after 24h growth at various concentrations of three types of supernatant. Supernatants were produced by incubating communities as in the main experiment in the antibiotic+plasmid treatment, the antibiotic+no plasmid treatment, and a control (incubated sterile medium with antibiotics). We prepared each type of supernatant by pooling 96 replicate microcosms (to obtain sufficient culture volume) and filtering (0.2 μm). We then measured growth of all seven strains (shown in separate panels) in pure culture at various concentrations of each supernatant type, diluted in fresh LB (*x*-axis, given as the percentage by volume of supernatant diluted in fresh LB medium). To compensate for the effect of nutrient depletion during supernatant preparation, which could potentially create a false signal of growth inhibition in downstream experiments, we added 10% and 5% of concentrated LB solution (at four times the standard concentration) to treatments with undiluted and 50% supernatant, respectively. This makes interpretation of quantitative growth scores in these treatments relative to other concentrations problematic, but enables us to rule out nutrient depletion as an explanation for zero growth of some species in these supernatants. Supernatant cultures were inoculated in 100 μl microcosms from overnight cultures by 1000-fold dilution as above, with three replicates in each combination. We estimated growth after 24 hours by Optical Density, OD (600 nm) with an Infinite M200 Pro NanoQuant Tecan plate reader (Männedorf, Switzerland). Note the different *y*-axis scale for *E. faecalis*, used because of the much lower data range for this species. The bars at each dilution for each treatment represent the mean (*n* = 4).

**
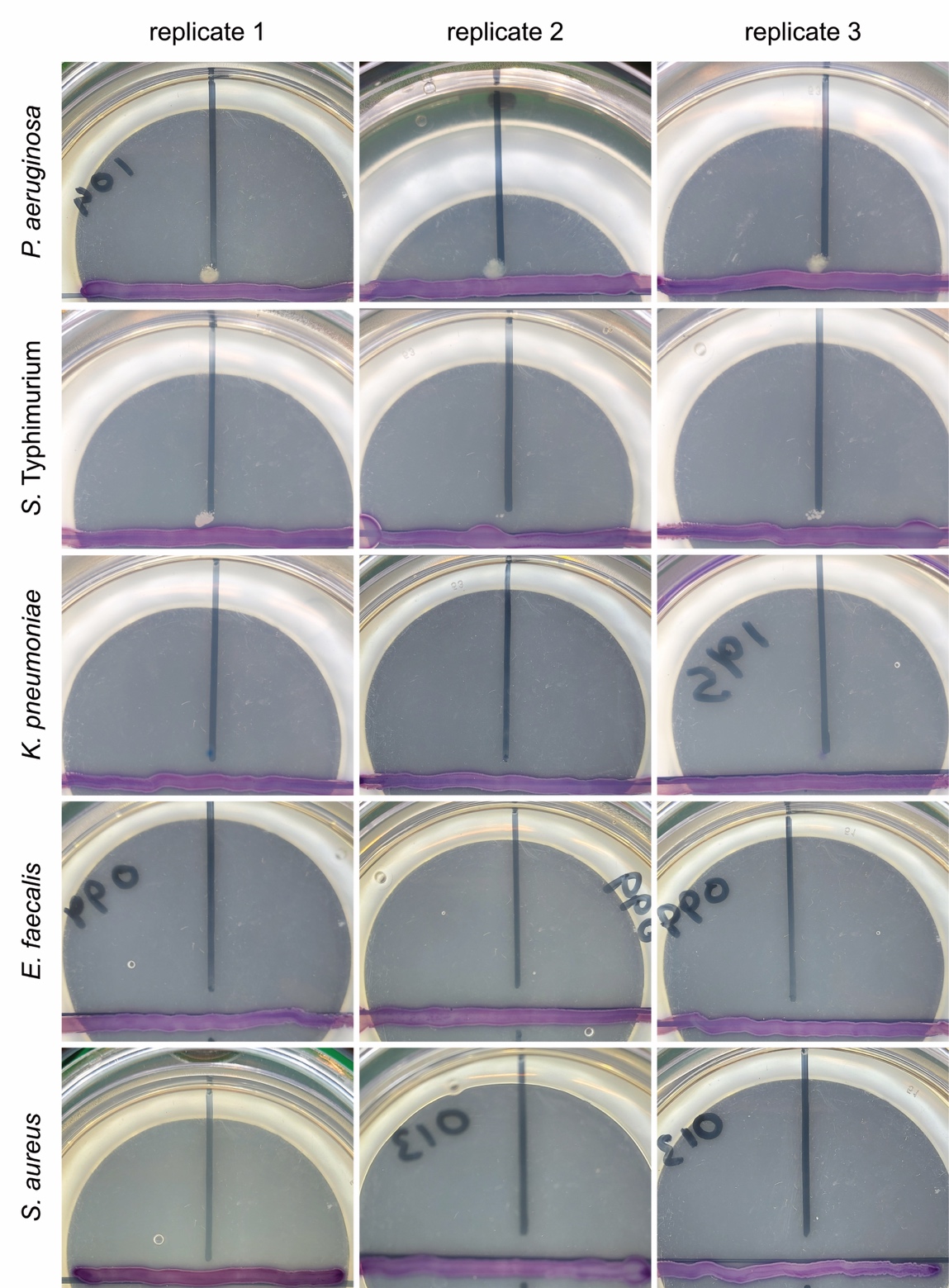
**

**Figure S4.** Two out of five antibiotic-susceptible species (*P. aeruginosa* and *S*. Typhimurium) show visible growth on antibiotic-agar in proximity to a resistant, plasmid-carrying *E. coli* strain. Each susceptible species (rows) was streaked on chromatic agar with antibiotics (down the vertical black line) perpendicular to the pOXA-48 carrying *E. coli* (the purple horizontal line of bacterial growth) and incubated for 24 hours. We made three plates per species (columns), leaving ~0.5 cm between the strains on each plate, checking after incubation for evidence growth of the susceptible strain was increased by proximity to the plasmid-carrying strain. Antibiotics were used at 7.5 μg/ml piperacillin and 1.5 μg/ml tazobactam. Each combination was replicated three times (the three columns in each row).

**
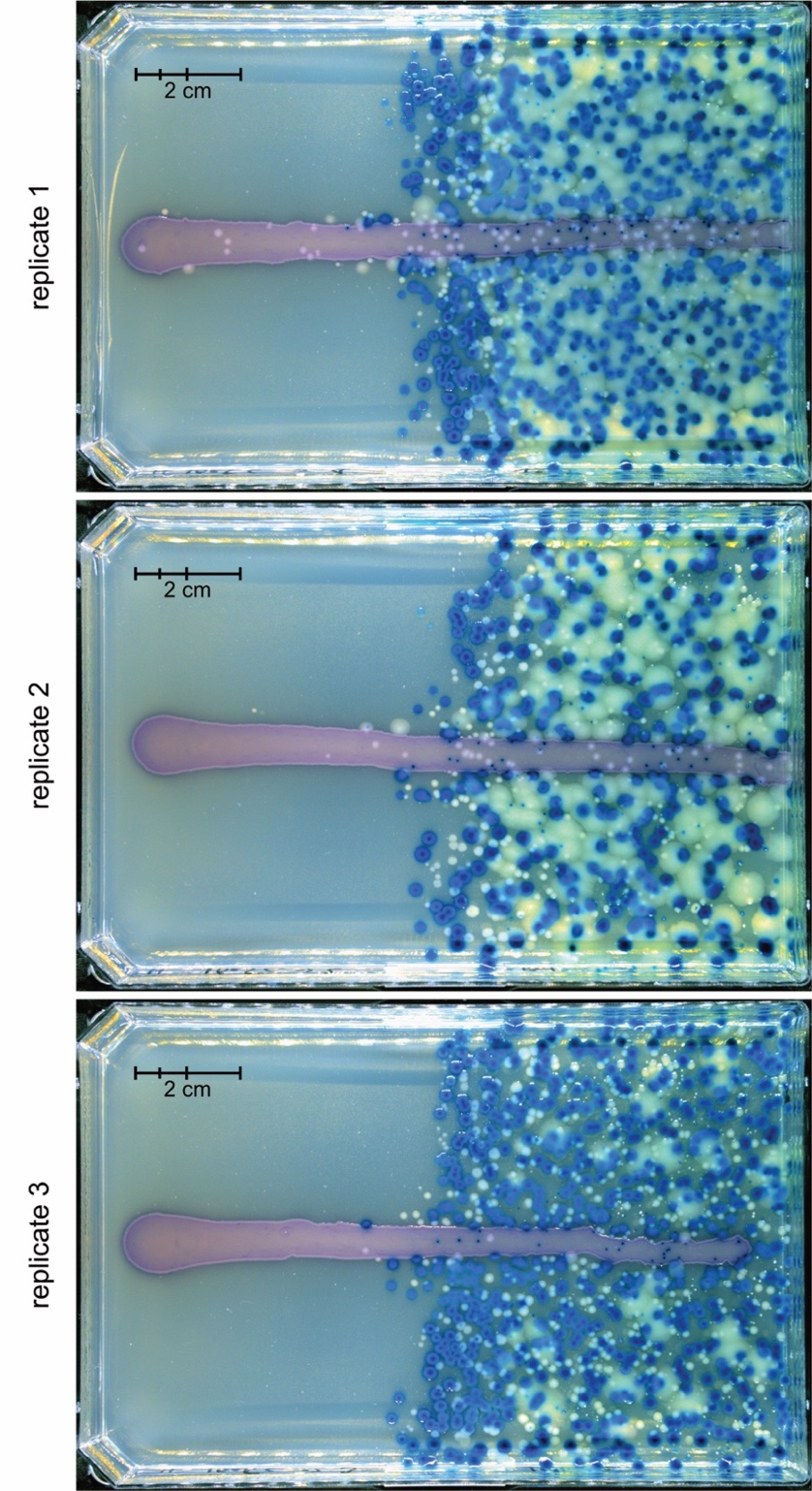
**

**Figure S5.** Multispecies communities plated on chromatic agar with a gradient of piperacillin+tazobactam (left-to-right; maximum concentration in overlay agar = 5 µg/ml piperacillin and 1 µg/ml tazobactam) with the plasmid-carrying *E. coli* strain streaked horizontally (purple). *S.* Typhimurium appears as defined, small off-white colonies, *P. aerugionosa* appears as diffuse off-white colonies, *K. pneumoniae* appears dark blue, *E. faecalis* appears light blue and *S. aureus* appears as small white colonies (see also Fig. 3B for labelling). To prepare gradient plates, prior to adding bacteria, we poured and dried a slanted slab of 20 ml LB agar containing 5 µg/ml piperacillin and 1 µg/ml tazobactam, before adding a further 20 ml antibiotic-free LB agar on top; we dried plates before adding bacteria.

**
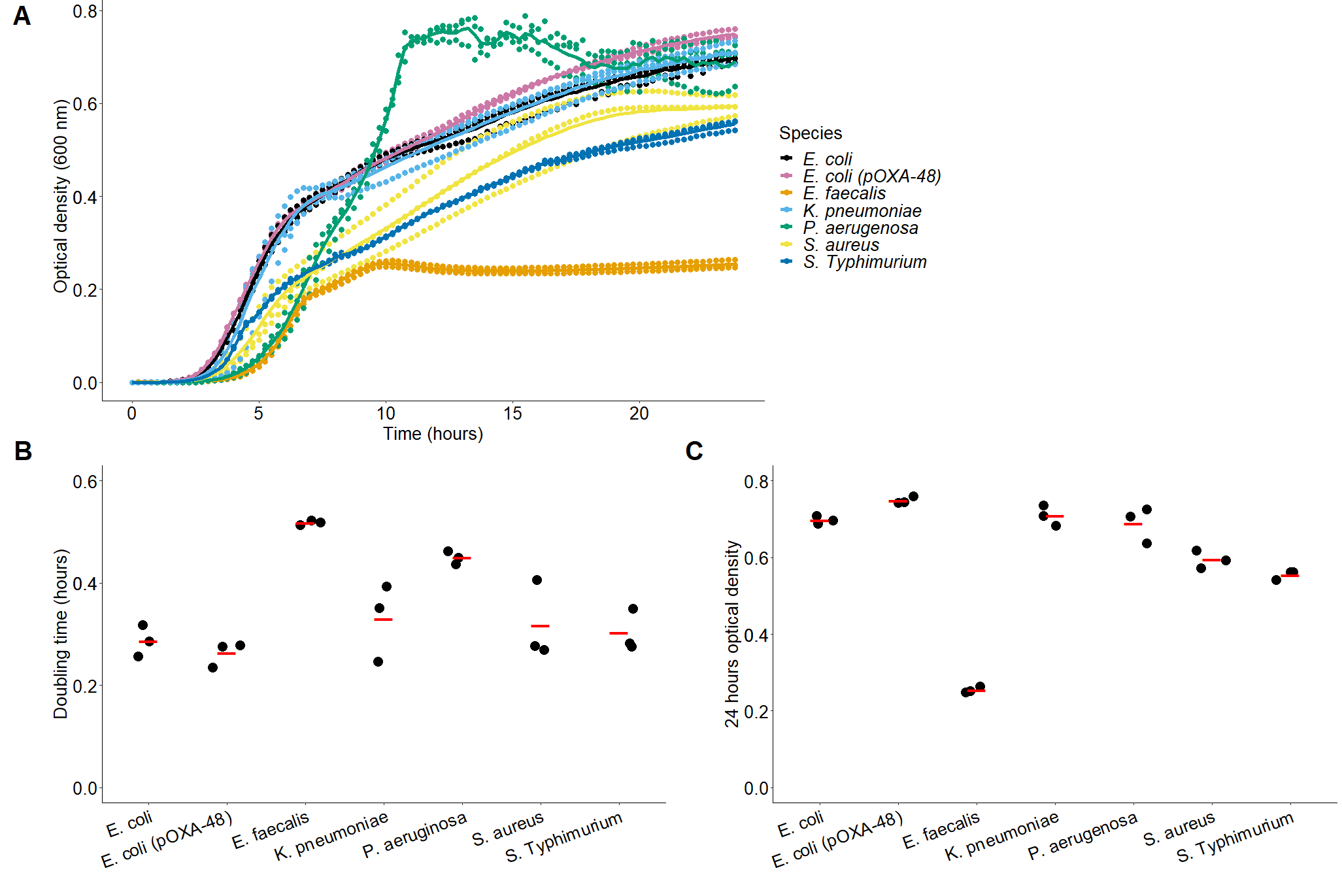
Figure S6**. Growth dynamics of all strains over 24 hours. **(A)** Abundance of each strain (see legend) measured by optical density over 24h. Lines show means, with three replicates per strain. **(B)** Growth rates estimated from the data shown in panel A, estimated using a sliding-window approach (see Methods). Average doubling time varied among strains (*F*_6,14_ = 12.4 and *p*<0.05 by one-way ANOVA); post-hoc Tukey’s HSD showed *E. faecalis* had a doubling time similar to that of *P. aeruginosa* and higher than all other strains, whereas *P. aeruginosa* had a doubling time not significantly different to that of *E. faecalis* and *K. pneumoniae* and higher than the other strains (*p* < 0.05). **(C)** Final abundances, inferred from final OD measurements after 24 hours for each species in panel A. Final abundance varied among species (*F*_6,14_ = 159.8 and *p* < 0.05 by one-way ANOVA); post-hoc Tukey’s HSD indicated *E. faecalis* had a lower average abundance than all other strains, *S. aureus* and *S*. Typhimurium were similar, significantly higher than *E. faecalis* and significantly lower than all other strains (*p* < 0.05).

**
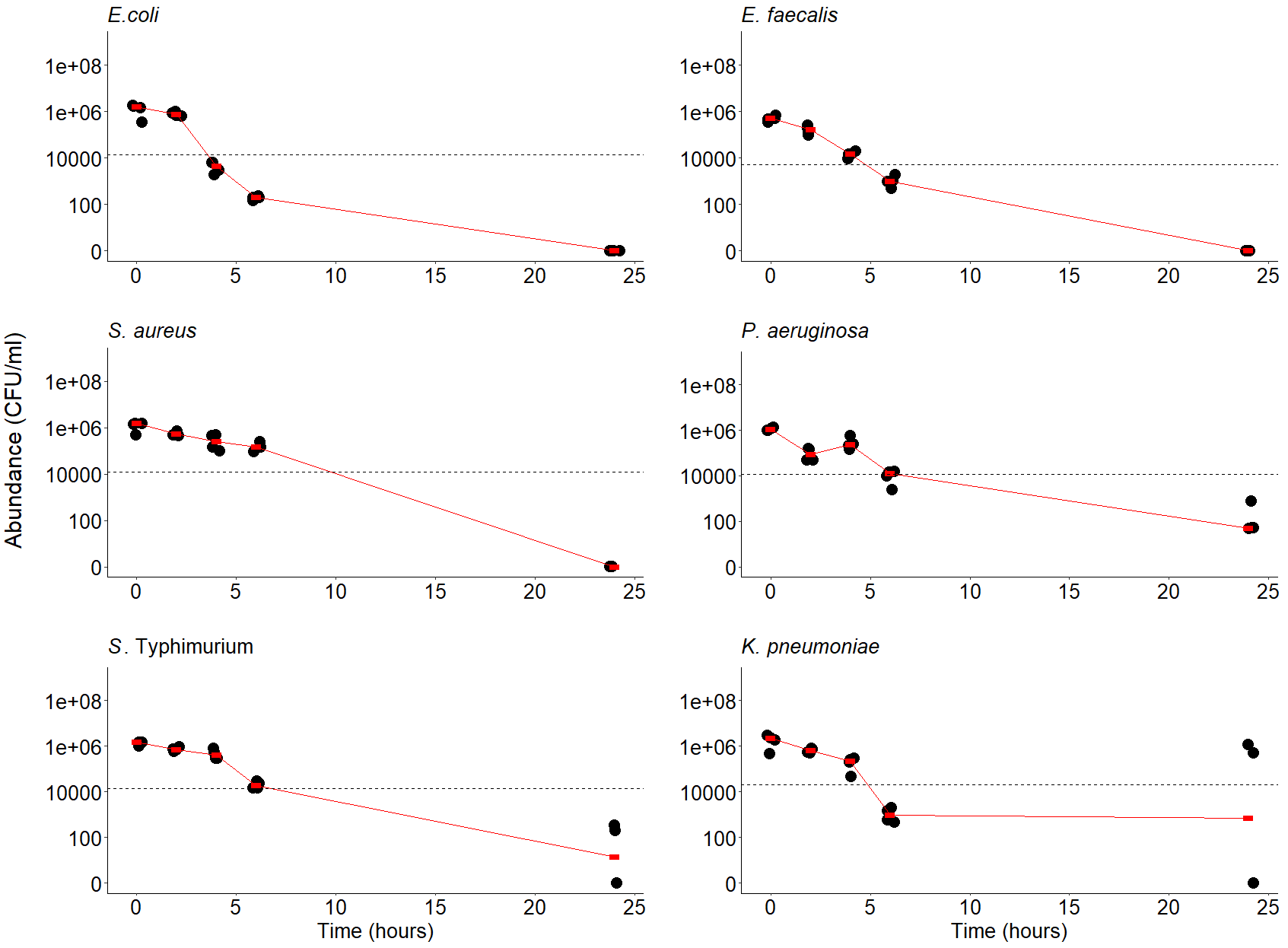
**

**Figure S7.** Survival of each susceptible species in pure cultures exposed to piperacillin+tazobactam over 24 hours. The same antibiotic concentrations were used as in the main experiment. Each point shows one replicate culture, and replicate cultures from different timepoints were independent from each other (each culture was sampled only once, by plating on agar without antibiotics). Different species responded differently to antibiotics over time (two-way ANOVA, Species × Time interaction: *F*_20,90_ = 2.6, *p* < 0.05). The horizontal dashed line in each panel shows 1% of the mean initial abundance for the relevant species (approximating the threshold for 99% killing); the red dash shows the median for each combination. Reduction in mean abundance by ≥99% was observed after 4 hours for *E. coli*, 6 hours for *P. aeruginosa*, *E. faecalis* and *K. pneumoniae*, and 24 hours for *S*. Typhimurium and *S. aureus*. For the two *K. pneumoniae* replicates with relatively high abundances after 24h, we suspected possible emergence of resistant mutants. We tested this by picking colonies from those replicates and testing their MICs as in the main text. We detected evidence of increased resistance in one of these two cultures, by an increase in MIC to 18.75 ug/ml piperacillin (compared to 9.375 ug/ml for the ancestral strain).

**References**

1. Baumgartner M, Bayer F, Pfrunder-Cardozo KR, Buckling A, Hall AR. Resident microbial communities inhibit growth and antibiotic-resistance evolution of *Escherichia coli* in human gut microbiome samples. PLOS Biology. 2020 Apr 20;18(4):e3000465.

2. Jacob AE, Hobbs SJ. Conjugal transfer of plasmid-borne multiple antibiotic resistance in *Streptococcus faecalis* var. zymogenes. J Bacteriol. 1974 Feb;117(2):360–72.

3. Holloway BWY 1955. Genetic recombination in *Pseudomonas aeruginosa*. Microbiology. 1955 Dec 1;13(3):572–81.

4. Stover CK, Pham XQ, Erwin AL, Mizoguchi SD, Warrener P, Hickey MJ, et al. Complete genome sequence of *Pseudomonas aeruginosa* PAO1, an opportunistic pathogen. Nature. 2000 Aug;406(6799):959–64.

5. Benz F, Huisman JS, Bakkeren E, Herter JA, Stadler T, Ackermann M, et al. Plasmid- and strain-specific factors drive variation in ESBL-plasmid spread in vitro and in vivo. ISME J. 2021 Mar;15(3):862–78.

6. León-Sampedro R, DelaFuente J, Díaz-Agero C, Crellen T, Musicha P, Rodríguez-Beltrán J, et al. Pervasive transmission of a carbapenem resistance plasmid in the gut microbiota of hospitalized patients. Nat Microbiol. 2021 May;6(5):606–16.

7. Simonsen L, Gordon DM, Stewart FM, Levin BR. Estimating the rate of plasmid transfer: an end-point method. J Gen Microbiol. 1990 Nov;136(11):2319–25.
